# Plant Proteomic Research 4.0: Frontiers in Stress Resilience

**DOI:** 10.3390/ijms222413362

**Published:** 2021-12-12

**Authors:** Sixue Chen, Setsuko Komatsu

**Affiliations:** 1 Department of Biology, Genetics Institute, University of Florida, Gainesville, FL 32611, USA; 2 Plant Molecular and Cellular Biology Program, University of Florida, Gainesville, FL 32610, USA; 3 Proteomics and Mass Spectrometry, Interdisciplinary Center for Biotechnology Research, University of Florida, Gainesville, FL 32610, USA; 4 Faculty of Environmental and Information Sciences, Fukui University of Technology, Fukui 910-8505, Japan

Large-scale high-throughput multi-omics technologies are indispensable components of systems biology in terms of discovering and defining parts of the system. Once the parts are functionally characterized, they become foundational for synthetic biology. Proteomics, as one of the essential omics in systems biology, provides the functional analysis of the expressed genome and generates detailed information that can be integrated with those obtained by other traditional and new omics approaches [1,2,3]. Systems biology and synthetic biology are the current frontiers of many areas of plant biology. As sessile organisms, plants are subject to varying environmental conditions, such as frequent changes in light exposure, humidity, temperature extremities, flooding, and other abiotic and biotic stresses, which can disturb and inhibit their growth and development [4,5]. These challenges to plants and crops are growing more severe as the effects of climate change become worse and increase the likelihood of inflicting extreme stresses on plants [4,6]. For example, the past seven years have been on track to be the warmest on record, according to the World Meteorological Organization (WMO) State of the Global Climate 2021 report [6]. In summer 2021, heatwaves in Canada and southwestern USA have pushed temperatures to almost 50 °C in a village in British Columbia and to 54.4 °C in California’s Death Valley. Many parts of the Mediterranean also experienced record temperatures. Accompanying the exceptional heat, drought and natural fires devastated communities and agriculture production. How plants respond to these environmental stresses, how crop production will be affected by climate change, and what strategies will be effective to boost crop stress resilience are fundamental questions for the plant research community.

Over the past two decades, plant proteomics has provided a deep knowledge and understanding of the diverse proteoforms and different plant processes owing to significant advancements in concepts, technologies, approaches, and platforms in plant proteomics. Proteome directly reflects proteotype, which is the proteomic state that uniquely underlies a phenotype [7]. Thus, proteotype connects genotype to phenotype, and it is affected by development and the environment. A proteome contains all the proteoforms, i.e., all the protein species that are expressed and modified, not just the genome-centric proteome. It has become clear that plant proteomics has moved from simply cataloging to profiling posttranslationally modified proteins (i.e., proteoforms), which include proteins with phosphorylation, acetylation, methylation, redox modifications, ubiquitinylation, glycosylation, etc. It has also moved from a 2D-gel-based approach to a gel-free liquid chromatography (LC)-based approach. In addition to traditional data-dependent acquisition (DDA), data-independent acquisition (DIA) has shown great utility and power in potentially achieving high proteome coverage and depth [8,9,10]. Thus, the amalgamation of diverse analytical techniques, complemented with genome-sequence data, modern bioinformatics tools, and improved sample preparation and fractionation strategies, provides an unprecedented opportunity to characterize novel proteins/proteoforms in spatial and temporal resolution and under different environmental conditions. This special issue of Plant Proteomic Research 4.0 captures the recent advancements in proteomics and addresses the current challenges of plant stress response and resilience in the ever-changing climate. It contains 12 articles, including 3 reviews and 9 original research articles.

The three reviews deal with pollen phosphoproteomics [11], starch biosynthesis-related proteins and posttranslational modifications (PTMs) in rice developing seeds [12], and PTMs of waxy proteins in rice grain [13]. Klodova and Fila [11] summarized phosphoproteomics studies in the past decade of male gametophyte developmental stages (mostly mature pollen grains) in different species (Arabidopsis, tobacco, maize, and kiwifruit). They highlighted common phosphoproteins in the different species and compared pollen phosphoproteomes with soybean root hair phosphoproteome to deduce shared mechanisms underlying the polarized tip growth of pollen tubes and root hairs. The next review by Tappiban et al. [12] provides a comprehensive analysis of proteins and PTMs involved in starch biosynthesis at different developmental stages of rice developing seeds. Several starch biosynthesis proteins were targeted by phosphorylation, acetylation, succinylation, lysine 2-hydroxyisobutyrylation, and malonylation. One enzyme, phosphoglucomutase, is commonly targeted by five different PTMs. The crosstalk and functions of these PTMs in starch biosynthesis are to be elucidated in future studies. The third review by Adegoke et al. [13] followed up with PTM of waxy protein (Wx), i.e., the granule-bound starch synthase, responsible for amylose biosynthesis. Wx is subject to phosphorylation modification, and a decrease in phosphorylation leads to low Wx activity and low amylose content. Employing PTM regulation of Wx may be effective in improving the yield of low-amylose rice to meet the market demand.

The nine research articles include three related to temperature, two on water stress, two on salt stress, one on fungal pathogens, and the last one on field-grown potato leaf apoplast proteome. Pang et al. [14] reported high-temperature stress on the phosphoproteomes of developing grains of two indica rice varieties with different starch qualities. The results revealed dynamic phosphorylation changes to starch biosynthesis enzymes in response to high-temperature stress, highlighting the importance of phosphorylation-mediated regulation in the biosynthesis of amylose and amylopectin, which determines the rice grain quality. Ginsawaeng et al. [15] employed both proteomics and gas chromatography–mass spectrometry-based metabolomics to reveal a heat-stable proteome and a highly connected late embryogenesis abundant (LEA) protein network during Arabidopsis seed germination. In the same vein of the heat-stable proteome, Sun et al. [10] are interested in understanding the proteomic basis of how sacred lotus maintains a stable floral chamber temperature under varied environmental temperatures between about 8 and 45 °C. They implemented a DIA-based quantitative proteomics approach to identify and quantify a total of 6913 proteins in lotus. A protein module highly related to the thermogenic phenotype was proposed to mainly involve metabolic processes, fatty acid degradation, and ubiquinone synthesis. Although high-temperature stress is different from drought stress, drought is often associated with high temperatures. San-Eufrasio et al. [16] aimed to identify proteins and proteotypic peptides as potential drought tolerance markers for holm oak using a label-free proteomics dataset of 4470 leaf proteins identified from oak seedlings from four different Andalusian populations in southern Spain. A total of 30 proteins and 46 derived peptides were selected as putative markers of drought tolerance in at least two populations. Among them, subtilisin and chaperone GrpE were increased under drought in three populations, thus they have a high potential of being the drought tolerance markers in oak trees. In addition to drought, climate change also brought irregular flooding [4,6]. Komatsu et al. [17] investigated molecular mechanisms of soybean flooding tolerance using gel-free/label-free proteomics with wild-type and flooding-tolerant mutants generated using gamma-ray irradiation. Although flooding stress affected proteins in both wild-type soybean and the mutant, those exhibiting opposite changes in abundance between the wild type and the mutant under flooding stress were interesting (e.g., calreticulin). Further analyses of alcohol dehydrogenase and glycoprotein profiles suggest that the mutant exhibits flooding tolerance through decreasing fermentation and enhancing glycoprotein folding, thereby minimizing cell death under flooding stress. Like the water stresses, salt stress poses a major negative impact on plant growth and agricultural production. Zhang et al. [18] analyzed endoplasmic reticulum (ER) proteomic changes in wheat leaves in response to salt stress using label-free proteomics. Of the 233 ER-localized differential proteins, salt stress significantly increased the levels of protein disulfide isomerase and heat shock proteins and decreased ribosomal proteins. Transcriptional regulation accounts for about half of the differential protein changes, highlighting the potential involvement of transcriptional factors in plant salt stress response. Zhang et al. [19] actually cloned a homeobox 7 (HB7) transcription factor important for salt tolerance of common ice plants. Overexpression of HB7 enhanced plant tolerance to 500 mM NaCl treatment. Label-free proteomics revealed that proteins increased in HB7-overexpression plants are involved in transport, catalytic activity, biosynthesis of specialized metabolites, and response to stimuli. Identifying the downstream targets of HB7 is an interesting direction towards understanding the mechanisms of HB7-mediated salt tolerance and potentially utilizing HB7 in marker-based crop breeding for improving salt tolerance. Tomatoes and potatoes are widely grown and consumed worldwide, especially in Europe, America, and Asian countries. However, late blight disease caused by *Phytophthora infestans* is a major challenge to the production of tomatoes and potatoes. Fan et al. [9] examined tomato leaf proteomic changes at different stages of *P. infestans* pathogenesis using DIA proteomics. A total of 6631 tomato proteins and 678 *P. infestans* proteins were profiled at three different timepoints of pathogenesis. Tomato proteins regulated by *P. infestans* during different phases of pathogenesis have functions in immunity, signaling, defense, and metabolism. The results provide an important resource for developing new strategies towards controlling late blight disease. Currently, most of the studies on plant response to abiotic and/or biotic stresses have been conducted under controlled laboratory conditions. Whether the results obtained can be translated into field conditions in agricultural practice is not known. Abreha et al. [20] took a big stride by investigating apoplastic proteomic changes of potato growing at two field sites in June to August across two years. The plants were also divided into fungicide treated groups and untreated control groups. Although no significant differences were observed between fungicide treated and untreated control samples, they did observe many differential apoplastic proteins in response to environmental or developmental factors in the two growing seasons. Using activity-based protein profiling, the differential activities of serine hydrolases and β-glycosidases revealed the seasonal effects. The apoplastic proteomics data obtained from the field-grown plants not only lay an important foundation for understanding the physiological state of crops grown under complex environmental conditions, but also bring up an important question of the translationability of growth chamber/greenhouse experiments. This work paves a new direction for agriculture-focused proteomics research.

In summary, the articles collected in this special issue of Plant Proteomic Research 4.0 reflect the current frontiers of plant proteomics, focusing on development and environmental factors. An obvious theme is environmental stresses, including temperature [10,14,15], drought [16], flooding [17], salt stress [18,19], pathogen [9], and agriculture field conditions [20]. Another topic is proteoforms/PTMs that several articles have focused on [11,12,13,14,16,17]. In addition, many studies are on crop species, e.g., tomato [9], maize [11], rice [12,13,14], soybean [17], wheat [18], and potato [20]. Last but not least, 2D gel-based proteomics seems to have become obsolete, with LC-based proteomics taking its place. It is great to see the application of DIA proteomics [9,10]. It will inspire more and more scientists to use this fairly new technology in addition to traditional DDA proteomics. The guest editors hope that this special issue will provide readers with a framework for understanding the status of plant proteomics and insights into new technologies and directions of proteomics in the frontiers of systems biology and synthetic biology. Due to time and space limitations, there are still many exciting developments in plant proteomics, e.g., PTM crosstalks, protein complexes, multi-omics, and single-cell/single-cell type proteomics that were not fully covered or included here. For sure, these new developments will be the subject of future special issues on plant proteomic research. Finally, the guest editors would like to express gratitude to all the authors for their contributions and the reviewers for their critical assessments of these articles. Additionally, they also want to thank the Assistant Editor, Ms. Dani Wu, for the opportunity to serve as guest editors for “Plant Proteomic Research 4.0”. They would highly appreciate your continuous support for future special issues.

## Data Availability

Not applicable.
